# Dysregulated responses to stress and weight in people with type 2 diabetes

**DOI:** 10.1016/j.jpsychores.2023.111354

**Published:** 2023-07

**Authors:** Ruth A. Hackett, Alessia Gareddu, Laura Panagi, Andrew Steptoe, Lydia Poole

**Affiliations:** a Health Psychology Section, Institute of Psychiatry, Psychology and Neuroscience, King's College London, London, UK; b Department of Psychiatry, School of Clinical Medicine, University of Cambridge, Cambridge, UK; c Behavioural Science and Health, Institution of Epidemiology and Health Care, University College London, London, UK; d Department of Psychological Interventions, School of Psychology, University of Surrey, Guildford, UK

**Keywords:** Laboratory stress testing, Obesity, Type 2 diabetes

## Abstract

**Objective:**

Dysregulated stress responsivity has been linked with weight gain in healthy samples. However, the relationship between disturbances in stress-related biology and changes in weight in people with type 2 diabetes (T2D) is unclear.

**Method:**

A total of 66 participants with T2D underwent laboratory stress-testing in 2011–2012. Cardiovascular, neuroendocrine and inflammatory responses to standardised mental stress were assessed, and Body Mass Index (BMI) was measured. Participants self-reported information on BMI in 2019. Associations between stress-related biological responses and BMI at follow-up were modelled using linear regression adjusting for age, sex, resting biological levels and baseline BMI.

**Results:**

Blunted diastolic blood pressure reactivity (*B* = −0.092, 95% CI -0.177; −0.007, *p* = 0.034) as well as poorer systolic blood pressure (*B* = −0.050, 95% CI -0.084; − 0.017, *p* = 0.004), diastolic blood pressure (*B* = −0.068, 95% CI -0.132; −0.004, *p* = 0.034) and heart rate (*B* = -0.122, 95% CI -0.015;-0.230, *p* = 0.027) recovery post-stress were associated with higher BMI 7.5 years later. Greater interleukin-1 receptor antagonist (*B* = 16.93, 95% CI 6.20; 27.67, *p* = 0.003) and monocyte chemoattractant protein-1 reactivity (*B* = 0.04, 95% CI 0.002; 0.084, *p* = 0.041) were associated with weight gain. No significant associations were detected for interleukin-6 or laboratory cortisol measures.

**Conclusion:**

Disturbances in stress-related biology may promote weight gain in people with T2D. Research with a larger sample size is required to explore associations between stress responsivity and BMI in people with T2D.

## Highlights

• Obesity is common in diabetes and increases the risk of diabetes-complications.
• We assessed stress responses and later body mass index (BMI) in diabetes.
• Blunted cardiovascular reactivity and poor heart rate recovery are linked with weight gain.
• Greater inflammatory responses are linked with higher BMI 7.5 years later.
• Disturbed stress-related biology may promote weight gain in diabetes.

## 1 Introduction

Obesity is involved in the pathogenesis of type 2 diabetes (T2D). Excess weight is a T2D risk factor [1] and obesity is more common in people with than without the condition [2]. Additionally, obesity increases the risk of diabetes-related complications [3]. One hypothesised contributor to the development and maintenance of obesity is psychological stress [4], as it influences functioning across multiple biological systems [5].

Acute laboratory stress trials assess dynamic biological responses to standardised tasks under controlled conditions [6]. Prospective relationships between stress responses and weight have been studied in healthy individuals. A study of 1276 Scottish participants associated blunted heart rate (HR) reactivity with future obesity [7]. This was replicated in a Dutch cohort [8]. However, not all studies have associated cardiovascular reactivity with weight change [9]. Little research has assessed cortisol or inflammatory responses and future weight. However, heightened cortisol [10] and inflammatory [11,12] reactivity has been linked with other health outcomes prospectively.

People with T2D report greater stress [13] and have altered biological stress responses compared with healthy controls [14]. One study linked inflammatory responses with future mental health in T2D [15]. However, the association of inflammatory responses with physical health in T2D is unclear. Therefore, in this exploratory study we investigated the impact of cardiovascular, neuroendocrine and inflammatory stress responses on weight in T2D. Specifically, we hypothesised that blunted cardiovascular responses, along with heightened cortisol and inflammatory responses to standardised laboratory stress tasks would be associated with greater body mass index (BMI) over 7.5 years follow-up.

## 2 Methods

### 2.1 Participants

In 2011/12, 140 participants with T2D participated in a laboratory stress trial [14]. Enrolment was limited to those who were free of coronary, inflammatory and mood disorders at baseline based on medical records and subjective reports. Details on the sample are reported elsewhere [14]. In 2019 (7.5 years later on average), they were invited to complete a postal questionnaire [15]. Ethical approval was granted by the National Research Ethics Service. Participants provided fully informed written consent.

### 2.2 Procedure

A detailed procedure is reported elsewhere [14,16]. HR, systolic and diastolic blood pressure (SBP; DBP) were monitored continuously using a Finometer and a venous cannula was inserted for blood sample collection. After a 30-min rest period, the baseline blood sample was drawn, and saliva was obtained using a Salivette for cortisol assessment. Data from the final 5 min of rest period were averaged to constitute baseline cardiovascular values. Then two 5-min stress tasks were administered. Averaged cardiovascular measures were taken during the task. Blood and saliva were collected immediately post-task. Further cardiovascular measures and blood samples were taken 45- and 75-min post-task. Cortisol samples were obtained at 20-, 45- and 75-min post-task. Assay procedures for interleukin-6 (IL-6), IL-1 receptor antagonist (IL-1RA), monocyte chemoattractant protein-1 (MCP-1) and cortisol are described elsewhere [[16], [17], [18]].

### 2.3 Mental stress tasks

Two standardised tasks were administered to induce stress [19]. The Stroop colour-word interference task requires repeated identification of target colour words printed in incongruous ink. The mirror tracing task involves tracing the outline of a star that can only be seen in mirror image. When the pen goes outside the line, a beep is emitted, and an error is counted.

### 2.4 Predictor variables: stress responses

Cardiovascular (i.e., SBP, DBP and HR) reactivity was assessed using a change score (mean task minus pre-task values), with higher values reflecting heightened reactivity and lower values reflecting blunted reactivity. Cardiovascular (i.e., SBP, DBP and HR) recovery was measured using 2 change scores: 1) mean task minus 45-min post-stress and 2) mean task minus 75-min post-stress, with lower scores reflecting slower recovery. Inflammatory responses were index using 3 change scores: 1) immediately post-task minus baseline, 2) 45-min post-task minus baseline and 3) 75-min post-task minus baseline, with larger scores reflecting increased reactivity. Cortisol responses were quantified using area under the curve, with larger values indicating greater responses [20].

### 2.5 Outcome variable: BMI

At baseline, height (cm) was assessed via stadiometer and weight (kg) using a Tanita scale. BMI was calculated (kg/m^2^). At follow-up (2019), BMI was calculated from self-reported weight and baseline height [15].

### 2.6 Covariates

Baseline age (years), sex (male/female) and pre-task values of the biological factor under consideration were included in all analyses. Prospective analyses additionally adjusted for baseline BMI.

### 2.7 Statistical analysis

The distribution of IL-6, IL-1RA and cortisol concentrations were skewed [[14], [15], [16]], so natural log transformation was applied (base of e ≈ 2.718). Descriptive characteristics are presented as means (standard deviations[SD]) and numbers (percentages). Non-logged values are presented in Table 1 for interpretative ease. Differences between participants who were retained and who were lost to follow-up were assessed using independent samples *t*-tests and Chi-square tests. Repeated measures analysis of covariance was used to assess changes in BMI between baseline and follow-up (two timepoints) adjusting for age and sex. Associations between stress responses and BMI were examined using multivariable linear regressions. Complete case analysis was conducted based on available data [15]. All analysed data are presented. Data are presented as unstandardised *B* coefficients with 95% confidence intervals (CIs). Analyses were conducted using SPSS v28.

**Table 1:** Participant characteristics (2011/2012).

	Baseline sample (*N* = 140)		Lost to follow-up (*n* = 74)		Retained sample (2019) (*n* = 66)	
Variable	N	Mean (SD) or n (%)	N	Mean (SD) or n (%)	N	Mean (SD) or n (%)
Age (years)	140	63.7 (7.0)	74	63.7 (7.4)	66	63.7 (6.7)
Sex (% men)	140	88 (62.9%)	74	49 (66.2%)	66	39 (59.1%)
Body mass index (kg/m^2^)	138	30.8 (5.7)	72	30.7 (6.1)	66	30.8 (5.3)
SBP baseline (mmHg)	136	126.1 (13.6)	71	126.3	65	125.9 (13.0)
SBP task (mmHg)	136	149.4 (20.6)	71	149.5	65	149.2 (21.7)
SBP 45-min (mmHg)	136	134.2 (20.3)	71	135.2	65	133.2 (19.0)
SBP 75-min (mmHg)	136	137.0 (17.0)	71	136.8	65	137.4 (16.9)
DBP baseline (mmHg)	136	71.7 (10.2)	71	71.2 (11.1)	65	72.4 (9.1)
DBP task (mmHg)	136	84.3 (12.5)	71	83.5 (13.2)	65	85.1 (11.8)
DBP 45-min (mmHg)	136	78.0 (14.9)	71	77.7 (15.8)	65	78.4 (13.9)
DBP 75-min (mmHg)	136	79.5 (13.8)	71	78.9 (14.1)	65	80.2 (13.6)
HR baseline (bpm)	138	71.8 (12.4)	72	70.7 (11.7)	66	73.0 (13.1)
HR task (bpm)	138	76.3 (12.4)	72	74.7 (12.2)	66	78.1 (12.2)
HR 45-min (bpm)	138	70.2 (12.2)	72	69.4 (12.2)	66	71.1 (12.3)
HR 75-min (bpm)	138	70.1 (11.9)	72	69.3 (11.7)	66	71.1 (12.3)
IL-6 baseline (pg/ml)	130	7.0 (2.2)	68	2.4 (1.5)	62	1.9 (1.0)*
IL-6 task (pg/ml)	126	6.8 (2.2)	67	2.4 (1.4)	59	2.0 (1.0)
IL-6 45-min (pg/ml)	115	8.0 (2.3)	61	2.6 (1.7)	54	1.9 (1.0)*
IL-6 75-min (pg/ml)	106	6.6 (2.3)	56	2.6 (1.5)	51	2.0 (0.9)*
IL-1RA baseline (pg/ml)	132	897.6 (549.4)	70	886.2 (477.6)	62	910.6 (624.4)
IL-1RA task (pg/ml)	128	884.1 (525.1)	69	893.4 (486.5)	59	873.1 (570.9)
IL-1RA 45-min (pg/ml)	117	859.9 (450.3)	63	902.9 (487.8)	54	809.9 (400.9)
IL-1RA 75-min (pg/ml)	107	818.3 (419.1)	58	853.3 (453.9)	49	777.0 (374.2)
MCP-1 baseline (pg/ml)	134	117.0	70	118.7 (33.6)	64	115.1 (35.8)
MCP-1 task (pg/ml)	130	114.9	69	118.5 (39.1)	61	110.9 (38.2)
MCP-1 45-min (pg/ml)	118	112.3	63	115.4 (37.8)	55	108.8 (32.8)
MCP-1 75-min (pg/ml)	108	108.7	58	110.0 (33.4)	50	107.2 (31.0)
Laboratory cortisol AUC (nmol/l)	127	671.2 (326.8)	67	694.4 (369.6)	60	645.3 (271.9)

AUC = Area under the curve; DBP = Diastolic blood pressure; HR = heart rate; IL-1RA = interleukin 1 receptor antagonist; IL-6 = interleukin 6; pg/ml = picogram per millilitre; MCP-1 = monocyte chemoattractant protein-1; mmHg = millimetres of mercury; nmol/L = nanomoles per litre; SBP = systolic blood pressure; SD = standard deviation; *p < 0.005.

## 3 Results

### 3.1 Participant characteristics

Sample characteristics are provided in Table 1. Further detail is available elsewhere [14,15]. Baseline participants were aged 50–75 years, most were male (62.9%) and obese (mean BMI = 30.75,SD = 5.82). Seventy-four (53%) participants were lost to follow-up. Participants with greater IL-6 pre-task (*t*(119.95) = −2.07, *p* = 0.041), 45-min (*t*(97.80) = −2.84, *p* = 0.006) and 75-min post-task (*t*(93.87) = −2.34, *p* = 0.021) were less likely to be retained. No other differences were observed between those retained and those lost to follow-up (*p* > 0.05). No significant changes in BMI (*F*(1,63) = 2.30, *p* = 0.366) over time ([mean = 30.79, Standard Error (SE) = 0.65] to [mean = 29.12, SE = 0.61]) were observed (*n* = 66).

### 3.2 Cross-sectional associations between stress responses and BMI

BMI was positively associated with pre-task values (Supplementary Table 1). No significant relationships were found between cardiovascular, IL-1RA, MCP-1 or cortisol responses and BMI (*p* ≥ 0.080; Table 2). IL-6 responses 75-min post-task were positively associated with BMI (*B* = 7.911, 95% CI 1.080;14.741, *p* = 0.024). No associations were found for IL-6 responses immediately or 45-min post-task (*p* ≥ 0.80). Cross-sectional findings were similar when restricting analyses to those retained at follow-up (Supplementary Table 2).

**Table 2:** Cross-sectional and prospective associations between laboratory responses to stress (2011/12) and Body Mass Index.

	Cross-sectional associations (2011/12)				Prospective associations (2019)			
	*n*	*B*	*95% CI*	*p*	*n*	*B*	*95% CI*	*p*
SBP task change from baseline (mmHg)	135	−0.042	−0.100; 0.016	0.156	65	−0.026	−0.061; 0.008	0.135
SBP 45-min change from task (mmHg)	135	−0.028	−0.075; 0.020	0.255	65	−0.025	−0.055; 0.005	0.097
SBP 75-min change from task (mmHg)	135	−0.010	−0.067; 0.046	0.720	**65**	**−0.050**	**−0.084; − 0.017**	**0.004**
DBP task change from baseline (mmHg)	135	−0.050	−0.181; 0.082	0.457	**65**	**−0.092**	**−0.177; −0.007**	**0.034**
DBP 45-min change from task (mmHg)	135	−0.046	−0.135; 0.042	0.299	65	−0.033	−0.095; 0.030	0.304
DBP 75-min change from task (mmHg)	135	−0.008	−0.100; 0.083	0.858	**65**	**−0.068**	**−0.132; −0.004**	**0.034**
HR task change from baseline (bpm)	137	−0.037	−0.230; 0.157	0.710	66	−0.112	−0.232; 0.007	0.065
HR 45-min change from task (bpm)	137	−0.013	−0.196; 0.170	0.887	66	−0.097	−0.022; 0.216	0.109
HR 75-min change from task (bpm)	137	−0.007	−0.190; 0.176	0.942	**66**	**−0.122**	**−0.230; −0.015**	**0.027**
IL-6 task change from baseline (ln)	124	6.353	−3.191; 15.896	0.190	59	0.314	−8.127; 8.756	0.941
IL-6 45-min change from baseline (ln)	113	8.189	−0.989; 17.367	0.080	54	0.893	−7.334; 9.119	0.828
IL-6 75-min change from baseline (ln)	**105**	**7.911**	**1.080; 14.741**	**0.024**	51	−3.662	−9.216; 1.891	0.191
IL-1RA task change from baseline (ln)	126	−2.369	−15.293; 10.555	0.717	59	5.510	−3.102; 14.121	0.205
IL-1RA 45-min change from baseline (ln)	115	3.912	−11.206; 19.030	0.609	**54**	**16.934**	**6.201; 27.668**	**0.003**
IL-1RA 75-min change from baseline (ln)	105	−0.160	−14.745; 14.425	0.983	**49**	**14.391**	**1.424; 27.357**	**0.030**
MCP-1 task change from baseline (pg/ml)	128	−0.015	−0.075; 0.044	0.610	**61**	**0.043**	**0.002; 0.084**	**0.041**
MCP-1 45-min change from baseline (pg/ml)	116	−0.015	−0.066; 0.035	0.554	55	−0.022	−0.070; 0.026	0.367
MCP-1 75-min change from baseline (pg/ml)	106	−0.003	−0.066; 0.060	0.924	50	0.016	−0.042; 0.075	0.580
Laboratory cortisol AUC (ln)	125	−0.001	−0.006; 0.003	0.515	60	−0.003	−0.006; 0.001	0.101

Complete case analysis was conducted based on available data. IL-6, IL-RA and cortisol data are log transformed.

All analyses adjusted for age, sex and baseline values of biological factor under study. Prospective analyses additionally adjusted for baseline Body Mass Index.

AUC = Area under the curve; CI = confidence interval; DBP = Diastolic blood pressure; HR = heart rate; IL-1RA = interleukin 1 receptor antagonist; IL-6 = interleukin 6; ln = log-n;, MCP-1 = monocyte chemoattractant protein-1; mmHg = millimetres of mercury; nmol/L = nanomoles per litre; pg/ml = picogram per millilitre; SBP = systolic blood pressure.

### 3.3 Prospective associations between stress responses and BMI

Blunted DBP reactivity (*B* = −0.092, 95% CI -0.177;-0.007, *p* = 0.034) and poorer (i.e., slower) SBP (*B* = -0.050, 95% CI -0.084;-0.017, *p* = 0.004), DBP (*B* = -0.068, 95% CI -0.132;-0.004, *p* = 0.034) and HR recovery 75 min post-task (*B* = -0.122, 95% CI -0.230; −0.015, *p* = 0.027) were associated with higher future BMI, independent of age, sex, baseline cardiovascular values and baseline BMI (Table 2). IL-RA responses 45-min (*B* = 16.93, 95% CI 6.20;27.67, *p* = 0.003) and 75-min post-task (*B* = 14.39, 95% CI 1.42;27.36, *p* = 0.030) were positively associated with follow-up BMI. Greater MCP-1 responses immediately post-task (*B* = 0.04, 95% CI 0.002;0.084, *p* = 0.041) were associated with weight gain. No associations were detected for IL-6 or cortisol (*p* ≥ 0.101).

## 4 Discussion

This study assessed associations between stress responses and weight in T2D. We found a positive cross-sectional association between IL-6 responses 75 min post-task and BMI. No other significant cross-sectional relationships were observed. In prospective analyses, blunted DBP reactivity and slower SBP, DBP and HR recovery were associated with higher BMI 7.5 years later. IL1-RA and MCP-1 reactivity were positively associated with later BMI. These findings were independent of baseline BMI. No significant prospective associations were detected for IL-6 or cortisol.

No previous study has investigated stress responses and weight in T2D. Cross-sectionally, we observed a positive association between IL-6 responses and BMI. Elevated resting IL-6 levels and adipose tissue inflammation have been observed in T2D [21]. Our finding adds to this work, suggesting that greater inflammatory reactivity is associated with BMI in T2D, independent of pre-task values.

Blunted DBP reactivity was linked with future BMI in our sample. Earlier work has associated blunted HR reactivity with future obesity in healthy participants [7,8]. Under allostatic load theory, blunted stress reactivity is considered a departure from normal functioning and health damaging [22]. Our study suggests blunted DBP may negatively impact future health in T2D. We associated poorer SBP, DBP and HR stress recovery with future BMI. People with T2D have slower cardiovascular stress recovery compared with healthy participants [14]. Our results suggest this poor recovery profile may promote weight gain in T2D.

IL-1RA and MCP reactivity was positively associated with future BMI in our study. We are unaware of other prospective work linking inflammatory responses and BMI in T2D. However, inflammatory reactivity has been linked with future hypertension and endothelial dysfunction in healthy samples [11,12]. We did not detect a prospective association between IL-6 and BMI. Resting IL-6 concentrations have not been consistently related to future weight [23]. As IL-6 has both pro- and anti-inflammatory effects, its relationship with weight is complex [24]. It is possible that different forms of inflammatory reactivity may be differentially associated with outcomes in T2D. Little research has assessed cortisol responses to stress and future BMI, and we did not detect an association here. Changes in diurnal cortisol output have been implicated in the pathogenesis of both obesity [25] and T2D [26] in observational studies. While interrelationships between basal cortisol, chronic stress and components of the metabolic syndrome such as high adiposity and insulin resistance have been observed [27], it is possible that cortisol responses to stress elevate disease risk through other pathways [10]. Research is required to test this notion.

## 5 Limitations

The study benefitted from a longitudinal design and the use of a standardised stress protocol. However, it was underpowered, with a small sample size and a high dropout rate [15]. Participants who were lost to follow-up had greater IL-6 levels at baseline than those who were retained in the study. Evidence suggests those who are lost to follow-up are often less healthy than those who are retained [28]. Thus, it is plausible in this study that non-responders with higher levels of inflammation had poorer outcomes than participants who provided follow-up data. Recruitment took place in the London area and most of the participants were of white ethnicity. Therefore, it is unclear whether these findings are generalisable to other groups. BMI at follow-up was self-reported and on average did not change significantly between assessment points but trended towards weight loss rather than gain. This may reflect mis-reporting or weight fluctuations over time [29]. We were only able to include a small number of covariates in our models due to the sample size. We did not include diet or physical activity measures though these influence stress-related biology [30,31]. Similarly, although diabetes severity (e.g., diabetes complications) and diabetes control (e.g., glycated haemoglobin; HbA1c) likely play a role in the association between stress responses and BMI, these were not included in our models.

Overall, disturbed stress responses may promote weight gain in T2D. Weight loss is beneficial for diabetes outcomes [3] and may alter stress responsivity [32,33]. Whether weight loss could attenuate the link between dysregulated stress responsivity and future BMI in T2D is unknown. Further study with a larger sample size is required to explore associations between stress responsivity and BMI in people with T2D.

## Funding

This work was supported by the 10.13039/501100000274British Heart Foundation (Grant RG/10/005/28296).

## Declaration of Competing Interest

None.

## Appendix A Supplementary data

Supplementary Table 1. Unadjusted associations between resting biomarker levels (2011/12) and Body Mass Index (2011/12) Supplementary Table 2. Cross-sectional associations between laboratory responses to stress and Body Mass Index (2011/12) in those who provided follow-up dataFootnoteView Edit Log304 Image 1
