# Supplementary material for: Dysregulated responses to stress and weight in people with type 2 diabetes
Source: J Psychosom Res. 2023 Jul;170:111354. doi: 10.1016/j.jpsychores.2023.111354 (PMC10758837; doi:10.1016/j.jpsychores.2023.111354)
Supplement: Supplementary file 1 — Supplementary Table 1. Unadjusted associations between resting biomarker levels (2011/12) and Body Mass Index (2011/12) Supplementary Table 2. Cross-sectional associations between laboratory responses to stress and Body Mass Index (2011/12) in those who provided follow-up dataFootnoteView Edit Log304 [file mmc1.docx]

| Supplementary Table 1. Unadjusted associations between resting biomarker levels (2011/12) and Body Mass Index (2011/12) | | | | |
| --- | --- | --- | --- | --- |
|  | *n* | *B* | *95% CI* | *p* |
| SBP baseline (mmHg) | 136 | 0.036 | -0.034; 0.105 | 0.312 |
| DBP baseline (mmHg) | 136 | 0.103 | 0.005; 0.200 | **0.040** |
| HR baseline (bpm) | 138 | 0.111 | 0.037; 0.186 | **0.004** |
| IL-6 baseline (ln) | 130 | 7.815 | 4.401; 11.228 | **0.001** |
| IL-1RA baseline (ln) | 132 | 14.867 | 11.624; 18.110 | **0.001** |
| MCP-1 baseline (pg/ml) | 134 | 0.028 | 0.001; 0.055 | **0.043** |
| Laboratory cortisol baseline (ln) | 136 | 1.436 | -2.771; 5.642 | 0.501 |
| Complete case analysis was conducted based on available data. IL-6, IL-RA and cortisol data are log transformed.  All analyses adjusted for age and sex.  CI= confidence interval; DBP= Diastolic blood pressure; HR=heart rate; IL-1RA= interleukin 1 receptor antagonist; IL-6= interleukin 6; ln= log-n, MCP-1= monocyte chemoattractant protein-1; mmHg= millimetres of mercury; pg/ml = picogram per millilitre; SBP= systolic blood pressure | | | | |

| Supplementary Table 2. Cross-sectional associations between laboratory responses to stress and Body Mass Index (2011/12) in those who provided follow-up data | | | | |
| --- | --- | --- | --- | --- |
|  | *n* | *B* | *95% CI* | *p* |
| SBP task change from baseline (mmHg) | 65 | -0.066 | -0.146; 0.014 | 0.105 |
| SBP 45-minute change from task (mmHg) | 65 | -0.037 | -0.108; 0.035 | 0.309 |
| SBP 75-minute change from task (mmHg) | 65 | -0.039 | -0.123; 0.045 | 0.358 |
| DBP task change from baseline (mmHg) | 65 | -0.183 | -0.373; 0.007 | 0.058 |
| DBP 45-minute change from task (mmHg) | 65 | 0.006 | -0.139; 0.151 | 0.933 |
| DBP 75-minute change from task (mmHg) | 65 | 0.029 | -0.123; 0.181 | 0.703 |
| HR task change from baseline (bpm) | 66 | -0.013 | -0.288; 0.261 | 0.923 |
| HR 45-minute change from task (bpm) | 66 | -0.024 | -0.295; 0.247 | 0.860 |
| HR 75-minute change from task (bpm) | 66 | 0.070 | -0.179; 0.319 | 0.576 |
| IL-6 task change from baseline (ln) | 59 | 0.525 | -16.891; 17.941 | 0.952 |
| IL-6 45-minute change from baseline (ln) | 54 | -0.194 | -16.998; 16.609 | 0.982 |
| IL-6 75-minute change from baseline (ln) | 51 | 0.653 | -10.819; 12.125 | 0.909 |
| IL-1RA task change from baseline (ln) | 59 | -5.373 | -22.265; 11.323 | 0.526 |
| IL-1RA 45-minute change from baseline (ln) | 54 | 2.527 | -18.992; 24.046 | 0.814 |
| IL-1RA 75-minute change from baseline (ln) | 49 | -3.326 | -28.584; 21.931 | 0.792 |
| MCP-1 task change from baseline (pg/ml) | 61 | 0.023 | -0.075; 0.122 | 0.640 |
| MCP-1 45-minute change from baseline (pg/ml) | 55 | -0.029 | -0.133; 0.076 | 0.585 |
| MCP-1 75-minute change from baseline (pg/ml) | 50 | -0.010 | -0.136; 0.116 | 0.875 |
| Laboratory cortisol AUC (ln) | 60 | -0.003 | -0.010; 0.005 | 0.514 |
| Complete case analysis was conducted based on available data. IL-6, IL-RA and cortisol data are log transformed.  All analyses adjusted for age, sex and baseline values of biological factor under study.  AUC= Area under the curve; CI= confidence interval; DBP= Diastolic blood pressure; HR=heart rate; IL-1RA= interleukin 1 receptor antagonist; IL-6= interleukin 6; ln= log-n; MCP-1= monocyte chemoattractant protein-1; mmHg= millimetres of mercury; pg/ml = picogram per millilitre; SBP= systolic blood pressure | | | | |
